# Protein Biomarkers of Bovine Defective Meats at a Glance: Gel-Free Hybrid Quadrupole-Orbitrap Analysis for Rapid Screening

**DOI:** 10.1021/acs.jafc.1c02016

**Published:** 2021-06-25

**Authors:** Enrique Sentandreu, Claudia Fuente-García, Olga Pardo, Mamen Oliván, Núria León, Noelia Aldai, Vicent Yusà, Miguel A. Sentandreu

**Affiliations:** † Instituto de Agroquímica y Tecnología de Alimentos (IATA-CSIC). Calle Agustín Escardino 7, 46980 Paterna, Valencia, Spain; ‡ Lactiker Research Group, Department of Pharmacy and Food Sciences, University of the Basque Country (UPV/EHU), Paseo de la Universidad 7, Vitoria-Gasteiz 01006, Spain; § Foundation for the Promotion of Health and Biomedical Research of the Valencia Region, FISABIO-Public Health, Av. Catalunya, 21, 46020 Valencia, Spain; ∥ Analytical Chemistry Department, University of Valencia, Edifici Jeroni Muñoz, Dr. Moliner 50, 46100 Burjassot, Spain; ⊥ Servicio Regional de Investigación y Desarrollo Alimentario (SERIDA), Carretera de Oviedo, s/n, 33300 Villaviciosa, Asturias, Spain; # Public Health Laboratory of Valencia, Av. Catalunya, 21, 46020 Valencia, Spain

**Keywords:** high-resolution proteomics, meat biomarkers, meat quality assessment, rapid proteomic screening, pre-slaughter stress

## Abstract

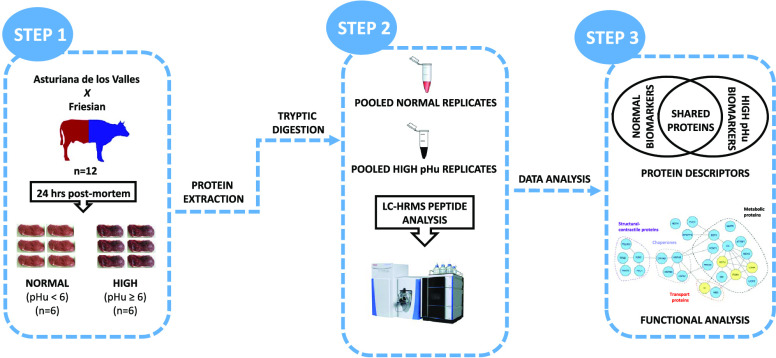

An understanding of biological mechanisms that could be involved in the stress response of animal cattle prior to slaughter is critical to create effective strategies aiming at the production of high-quality meat. The sarcoplasmic proteome of directly extracted samples from normal and high ultimate pH (pHu) meat groups was studied through a straightforward gel-free strategy supported by liquid chromatography hybrid quadrupole-Orbitrap high-resolution mass spectrometry (LC-HRMS) analysis. A stepped proteomic pipeline combining rapid biomarker hunting supported by qualitative protein Mascot scores followed by targeted label-free peptide quantification revealed 26 descriptors that characterized meat groups assayed. The functional study of the proposed biomarkers suggested their relevant role in metabolic, chaperone/stress-related, muscle contractility/fiber organization, and transport activities. The efficiency, flexibility, rapidity, and easiness of the methodology proposed can positively contribute to the creation of innovative proteomic alternatives addressing meat quality assessment.

## Introduction

Consumers are currently worried about ethics of food production concerning the implementation of animal welfare policies, greatly influencing their final decision on product selection.^1^ In this regard, the European Union is leading the promotion of animal well-being actions as a way to achieve sustainable development in the production of human foodstuffs.^2^ Pre-slaughter stress (PSS) is one of the most relevant issues among different conditioning factors related to animal care that can greatly affect the quality of meat, causing the occurrence of defective dark, firm, and dry (DFD) meat that is normally characterized by an ultimate pH (pHu) ≥ 6.0. Food authorities consider that pHu values higher than 6.0 at 24 h postmortem are intimately associated with PSS animals^3^ and DFD meats.^4^ Therefore, early detection of high pHu meats in the food chain is critical for the industry^5^ since defective raw material causes significant economic losses.^6^ Unfortunately, the value of pHu assessment is compromised since high values do not necessarily guarantee the presence of true DFD meats,^6^ requiring new diagnostic strategies. The efficiency of proteomic research for the hunting of predictive high pHu protein biomarkers stands up as a novel approach to discriminate meat from normal and PSS animals.^7−11^ However, results achieved to date strongly relied on gel-based methodologies that provided an excellent resolving power while still having important constraints such as the limited number of identified protein biomarkers and inaccurate quantitative results. Furthermore, the application of such approaches can be hindered by current trends in green analytical chemistry concerning the use of hazardous and nonsustainable chemicals (i.e., acrylamide) and excessive energy consumption by merging two protein purification steps.^12^

Nowadays, the development of fast, efficient, sustainable, and straightforward proteomic strategies is highly demanded for meat quality assessment. As an affordable gel-free approach for the discovery of peptide biomarkers, Sentandreu et al.^13^ proposed the use of OFFGEL fractionation followed by liquid chromatography-tandem mass spectrometry (LC-MS/MS) analysis featured by a conventional exploratory detector (three-dimensional ion trap, 3D-IT) for simultaneous qualitative/quantitative analysis of the bovine proteome. Although this accessible solution can be readily incorporated by industry, the use of low-resolution mass spectrometry (LC-LRMS) detection restricted the research to only noticeably abundant peptides, having additional limitations such as the inclusion of the OFFGEL fractionation step that extended the sampling procedure. Such drawbacks can be overtaken by liquid chromatography high-resolution mass spectrometry (LC-HRMS) analysis considering the sensitivity and high-output capacity of current devices. Moreover, minimal uncertainties achieved by HRMS detection favor simplification of sample preparation through appropriate background discrimination.

Regarding innovative analytical alternatives aiming at the development of efficient holistic proteomic approaches requested by system biology studies, LC-HRMS analysis perfectly fits routine and basic research expectations by minimizing the uncertainty of determinations.^14^ However, implementation of LC-HRMS technology in meat quality assessment occurred in very recent years considering mainly bottom-up proteomic studies for food authentication and the detection of adulterations in processed protein-based foodstuffs.^15^ In contrast to sensitive protein analysis commonly supported by targeted selected/multiple reaction monitoring (SRM/MRM) approaches,^16^ there are a limited number of proteomic alternatives carrying out the simultaneous qualitative and quantitative research of PSS biomarkers by LC-HRMS analysis. Recent studies supported by state-of-the-art Orbitrap^R^ and ion mobility time-of-flight (TOF) technologies successfully determined proteins linked to pHu in bovine meat.^5,17,18^ Nevertheless, implementation of these efficient strategies can be discouraging by the complex sampling procedures proposed, such as double trypsin digestion, preliminary desalting, molecular weight cutoff ultrafiltration, sample reduction, protein separation by bidimensional chromatography, and/or performing a subproteome assay restricted to a particular cell organelle (mainly mitochondrion). Thus, innovative approaches are needed to develop rapid, easy, and high-output LC-MS methodologies for the hunting of PSS proteomic predictors that can be suitable for both general overview and more targeted routine analyses in meat quality research.

This work aims to demonstrate the usefulness of a straightforward qualitative and quantitative LC-HRMS methodology for the rapid screening of protein biomarkers linked to meat quality. Direct analysis of sarcoplasmic protein extracts from normal and high pHu meat groups was studied by hybrid quadrupole-Orbitrap analysis. Preliminary protein characterization followed by targeted peptide quantitative analysis led to the tentative identification of potential meat biomarkers. Functional analysis and study of the interaction network of the proposed protein descriptors facilitated the understanding of different biochemical pathways that could be involved in the PSS response. The simplicity and high efficiency of this methodology can facilitate its easy implementation in multipurpose activities addressing rapid meat quality assessment.

## Materials and Methods

### Reagents and Solvents

LC-MS grade acetonitrile (ACN), formic acid (FA), ethylenediaminetetraacetic acid (EDTA, 99% purity), Tris buffer (99% purity), and 0.45 μm PVDF filters were from Scharlab (Scharlab S. L., Barcelona, Spain). Ultrapure grade water was from Millipore (EMD Millipore Co., Billerica, MA). Sucrose, protease inhibitor cocktail (P8340), and ammonium bicarbonate were from Sigma-Aldrich (Sigma-Aldrich Co., St. Louis, MO). Modified trypsin was from Promega (Promega, Madison, WI).

### Sample Preparation

Meat samples from 12 crossbred animals belonging to Asturiana de los Valles *x* Friesian breed were collected from a commercial abattoir in northeastern Spain. Muscle samples were from *Longissimus thoracis et lumborum* (LTL) of yearling bulls slaughtered at 14–15 months of age according to EU regulations (Council Regulation (EC) No. 853/2004 and No. 1099/2009). At 24 h postmortem, 10 g of the LTL muscle was excised from the 13th rib, and the epimysium was dissected. Meat samples were immediately vacuum-packed and stored at −80 °C until processed for protein extraction. Muscle samples were classified into two different groups according to their pHu values: normal pHu samples (NORMAL, *n* = 6) with pHu values below 6.0 (in our case: 5.53 ± 0.14) and high pHu samples (HIGH, *n* = 6) with pHu values higher than 6.0 (in our case: 6.56 ± 0.25). Determination of pH was performed at the sixth rib of the LTL muscle at 24 h postmortem.

Sarcoplasmic proteins were extracted according to Fuente-García et al.^9^ Briefly, half a gram of the muscle sample was homogenized in 4 mL of extraction buffer (10 mM Tris pH 7.6 containing 0.25 M sucrose, 1 mM EDTA, and 25 μL of protease inhibitor cocktail), centrifuged at 20 000*g* for 20 min at 4 °C, and the supernatant was filtered through a 0.45 μm PVDF filter. One hundred microliters of each sample was mixed with 300 μL of chilled EtOH (containing 0.15% FA), vortexed (20 s), stored at −20 °C for 20 min, and centrifuged at 3600*g* for 30 min at 4 °C. The supernatant was discarded and the resulting pellet was completely desiccated in an SPD121P SpeedVac vacuum concentrator (Thermo Scientific, San Jose, CA). Digestion of dried samples was carried out by adding 15 μL of a sequencing grade modified trypsin solution at a 12.5 μg/mL concentration and mixed with 20 μL of 50 mM ammonium bicarbonate (pH 8.5), being the mixture incubated overnight at 37 °C with continuous gently shaking. Tryptic digests were vacuum-dried as previously described. The samples were resuspended in 80 μL of an aqueous 0.1% FA solution. Forty microliters of each biological replicate (*n* = 6) was pooled according to assayed groups (normal vs high), giving rise to a final value of 240 μL each. Pooled sample groups were spiked with 60 μL of the internal standard (IS) solution composed by a tryptic hydrolysate of an almond (Prunus dulcis) protein extract as described by Sentandreu et al.,^13^ centrifuged at 20 000*g* for 5 min, separately poured into LC vials and kept at −80 °C until LC-HRMS analysis.

The study of pooled sample groups aimed at the development of a straightforward methodology for the rapid screening of meat quality biomarkers. Although this procedure reduces biological differences of individuals (replicates), it increases the power to detect changes between the averaged samples (meat groups) formed. Despite the undesirable dilution effect that pooling can cause insensitivity of peptides from some low-abundant proteins, the advantages of this strategy in terms of robustness and time efficiency valorize its use for rapid biomarker hunting.

### LC-HRMS Analysis

Chromatographic analysis was performed on a Thermo Vanquish Flex UHPLC system equipped with a quaternary pump, a vacuum degasser, and an open autosampler with a temperature controller (Thermo Fisher Scientific, San José, CA). Chromatographic separation of tryptic peptides was performed on a 150 mm × 2.1 mm, 3 μm particle-size Luna Omega PS C18 column (Phenomenex Inc, Torrance, CA) with the following separation conditions: solvent A, water/FA (99.9:0.1); solvent B, ACN/FA (99.9:0.1); separation gradient, initially 0% B, held for 15 min, linear 0–20% B in 2 min, held for 4 min, 40% B in 0.1 min, held for 9.9 min, 100% B in 0.1 min, washing with 100% B for 9.9 min, 0% B in 0.1 min, and column equilibration for 54.9 min; total run time, 95 min; flow rate, 50 μL/min; and injection volume, 5 μL. Column flow was conducted into the MS system during the 1.2–90 min time range diverting the rest of the run time to waste. Autosampler and column temperatures were set at 10 and 25 °C, respectively.

Mass spectrometry analysis was carried out on a hybrid quadruple-Orbitrap Thermo Q Exactive detector equipped with a heated electrospray (H-ESI) source operating in positive ion mode (Thermo Fisher Scientific, Bremen, Germany). The samples were studied by merging full MS^1^ and data-dependent MS/MS (dd-MS^2^) analyses. A full description of HRMS detection conditions is detailed in the Supplementary File.

The LC-MS platform of analysis was controlled by a PC operating the Xcalibur v. 2.2 SP1.48 software package (Thermo Scientific, San Jose, CA).

### Qualitative Analysis of MS/MS Data (Protein Mapping)

Proteins were identified through interrogation of dd-MS^2^ data by a licensed Mascot v.2.7 search engine (www.matrixscience.com) loading UP9136_B-Taurus and NCBIprot databases with the following settings: enzyme, trypsin; no fixed or variable modifications but enabling the “Error tolerant” option; peptide tolerance (monoisotopic) was 6 ppm and 0.02 Da for full MS^1^ and MS/MS analyses, respectively; peptide charge, +1 to +4; and taxonomy restriction parameter, Mammalia. The samples were further interrogated by loading the NCBIprot database, indicating “viridiplantae, green plants” as a taxonomy for the elucidation of almond peptides used as IS. The decoy option was used to estimate false positive rates by means of a false discovery rate (FDR) threshold of 1%. Only those identifications that have a protein score derived from individual peptide ion scores indicating identity or extensive homology (*p* < 0.05) were considered as true protein identifications.

### Quantitative Analysis of MS^1^ Data (Label-Free Peptide Quantification)

Identified proteins from meat groups assayed were rapidly screened according to their individual Mascot protein score achieved by loading the UP9136_B-Taurus database. Assignments exclusively found in normal or high samples were immediately considered (primary biomarker candidates) for further quantitative MS^1^ analysis. Then, protein score ratios of the identified proteins shared by both meat groups were calculated as suggested by Sentandreu et al.^13^ regarding the utility of protein scores as a coarse indicator of their abundance. Only those protein pairs (secondary biomarker candidates) that have a minimum 2-fold change variation in their protein score ratios were considered. Both primary and secondary candidates populated the preliminary list of potential protein biomarkers (Table S3, discussed below). Label-free MS^1^ quantification of proteotypic peptides (with the maximum ion score) from suggested candidates helped to refine previous rough results just considering protein scores from Mascot analysis. Freely available MZmine 2 v.2.53 (http://mzmine.github.io/download.html) loading an in-house library (Table S1), listing the aforementioned characteristic peptides of preliminary protein candidates, processed MS^1^ data as indicated by Sentandreu et al.^13^ with some modifications. For better results, merged MS^1^–dd-MS^2^ raw data files needed demultiplexing (http://proteowizard.sourceforge.net/) and only isolated MS^1^ information was handled for accurate quantification using the following optimized settings: mass tolerance, 5 ppm; minimum scans-across-peak (scan rate) for reliable quantification, 8;^19^ retention time tolerance for library interrogation, 0.5 min; and retention time tolerance for chromatograms alignment, 1 min. Chromatographic results were appropriately normalized through spiked IS and peptide ratios from meat groups assayed (normal/high or high/normal) were finally conformed to elaborate the definitive list of protein biomarkers discussed in protein functional analysis.

### Protein Functional Analysis

Proposed protein biomarkers were classified considering their biological process (BP) and cellular component (CC) from Gene Ontology (GO) terms powered by AmiGO website (https://amigo.geneontology.org/amigo/), KEGG pathway, annotated keywords (UniProt database), and local network cluster found in functional enrichment analysis performed by STRING v.1.11.1 freeware (ELIXIR, Wellcome Genome Campus, Hinxton, Cambridgeshire, U.K., https://string-db.org). Protein–protein interaction strength among biomarkers studied was assessed by STRING analysis, selecting “*Bos Taurus*” as the organism to perform interrogations. The results were further processed by Cytoscape v.3.8.2 (https://cytoscape.org)^20^ to elucidate protein networks.

## Results and Discussion

Original LC-HRMS (both merged MS^1^–dd-MS^2^ and isolated MS^1^ experimental results in the mzXML format) and Mascot generic format (mgf) data files generated in this study are freely available at http://hdl.handle.net/10261/228237.

### Accuracy of High-Resolution MS/MS Data Interrogation for Protein Mapping

FDR and extensive homology identification constraints considered in this study (see the Materials and Methods) made the Mascot study propose those reliable tentative protein identifications that have a minimum individual ion score of 19 (Figures S1A and S1B for normal and high, respectively). Table S2 lists identified proteins from normal (cursive red) and high pHu (bold black) pooled samples according to identification constraints considered. Protein families will be mentioned throughout the text according to acronyms detailed in Table S2.

Since the search of biomarkers can be limited by uncertainties derived from LC-MS detection, this study focused on the acquisition of unambiguous results based on accurate HRMS analysis. As an example, Figure S2 shows the protein sequence coverage of protein families identified in meat groups assayed with either high or low protein scores achieved (GAPDH with a score of 523 and BIN1 with a score of 22 in the normal sample, Figure S2A,B, respectively, and HSPA8 with a score of 315 and DBI with a score of 43 in the high pHu sample, Figure S2C,D, respectively). Observed peptide mass deviations were below 5 ppm in most cases (see the ppm column and dispersion of mass accuracy error vs molecular weight in Figure S2). High mass accuracy allowed that in general, Mascot analysis could greatly reduce uncertainties through the proposal of a unique amino acid sequence per interrogated query (MS/MS fragmentation pattern from tryptic peptides), as shown in Figure S3 (S3a and S3b for peptides from GAPDH and BIN1 in the normal sample, respectively, and S3C and S3D for HSPA8 and DBI in the high sample, respectively). In addition to univocal amino acid sequence matches, some protein families detailed in Table S2 listed queries with different feasible possibilities. Their unambiguous amino acid sequence assignment was eased through the very noticeable score difference mostly found between the first and the rest of the proposed sequence alternatives (Figure S4), corresponding to a peptide from PYGM in the normal sample.

### Preliminary Elucidation of Protein Biomarkers from Meat Groups Assayed

A rapid elucidation of potential biomarkers was achieved through the elimination of those proteins from Table S2 that were shared by both meat groups assayed (Table S3A, 30 proteins). Very clearly, the number of specific protein biomarkers (previously mentioned as primary biomarker candidates) was significantly higher (24 vs 6) in the high pHu sample compared to its normal counterpart (bold black vs italic red assignments in Table S3A). The results can be understood considering previous studies in sarcoplasmic protein extracts detailing how high pHu meat from stressed animals has an altered proteome exhibiting characteristic proteins such as heat shock proteins and α-crystallin B.^9^ Furthermore, low protein scores shown by assignments listed in Table S3A (22–115 range) suggested their low abundance in the sarcoplasmic proteome of meat groups assayed. In contrast, most proteins found in both sample groups had similar Mascot scores and yielded score ratios up to a 1.5-fold difference (Table S2), suggesting their scarce relevance as discriminants. Interestingly, few shared species (AHNAK, HSPA8, HSPB1, LDHA, PGM1, and TF, previously mentioned as secondary biomarker candidates; Table S3B) showed remarkable differences in their abundance as suggested by their fold change variation (minimum Mascot score ratio of 2).

### MS^1^ Label-Free Quantitative Analysis to Test the Reliability of Preliminary Biomarker Elucidation

The usefulness of the protein score (isolated and rationed) as a coarse but rapid semiquantitative indicator was assessed by label-free MS^1^ quantification of proteotypic peptides from candidates listed in Tables S3A and S3B (a total of 36 peptides merged in Table S1). Table S4 shows the relative MS^1^ quantitative results of the targeted LC-MS analysis carried out in this research. From the initial 36 candidates, only DBI and ACAA2 were exclusive of the high pHu sample group (Table S4). The remaining 34 potential candidates were detected in both meat groups but with significant differences. As mentioned in the Materials and Methods section, a minimum rate of 8 scans/peak was considered for reliable quantitative purposes. Lower values can lead to positive protein identifications (unambiguous high-resolution MS/MS analysis) but with no reliable peptide quantifications (below limit of quantification, BLQ, in Table S4). In our case, most BLQ assignments were defined by only one scan, finding all of them in the 1–3 scanning rate range. Since scans-across-peak is correlated with peptide abundance, a pseudo-quantitative value of a 100-fold change was granted to normal/high or high/normal normalized ratios of peptides found at the BLQ level in one of the meat groups studied but not in the other (i.e., HSPA8 and PRDX6; Table S4). Despite its inaccuracy, this strategy eased the rapid confirmation of many protein candidates as reliable biomarkers. Finally, peptides robustly quantified in both meat groups (i.e., PGM1 and CRYAB; Table S4) enabled the accurate determination of their fold change variation. As a result, from the 36 potential biomarkers initially proposed by the coarse protein score approach (Table S3), label-free quantitative analysis certified 26 of them (Table 1) as robust descriptors that exhibited a minimum of 2-fold change variation (normalized peak area ratio of 2; Table S4) in meat groups studied.

**Table 1:**
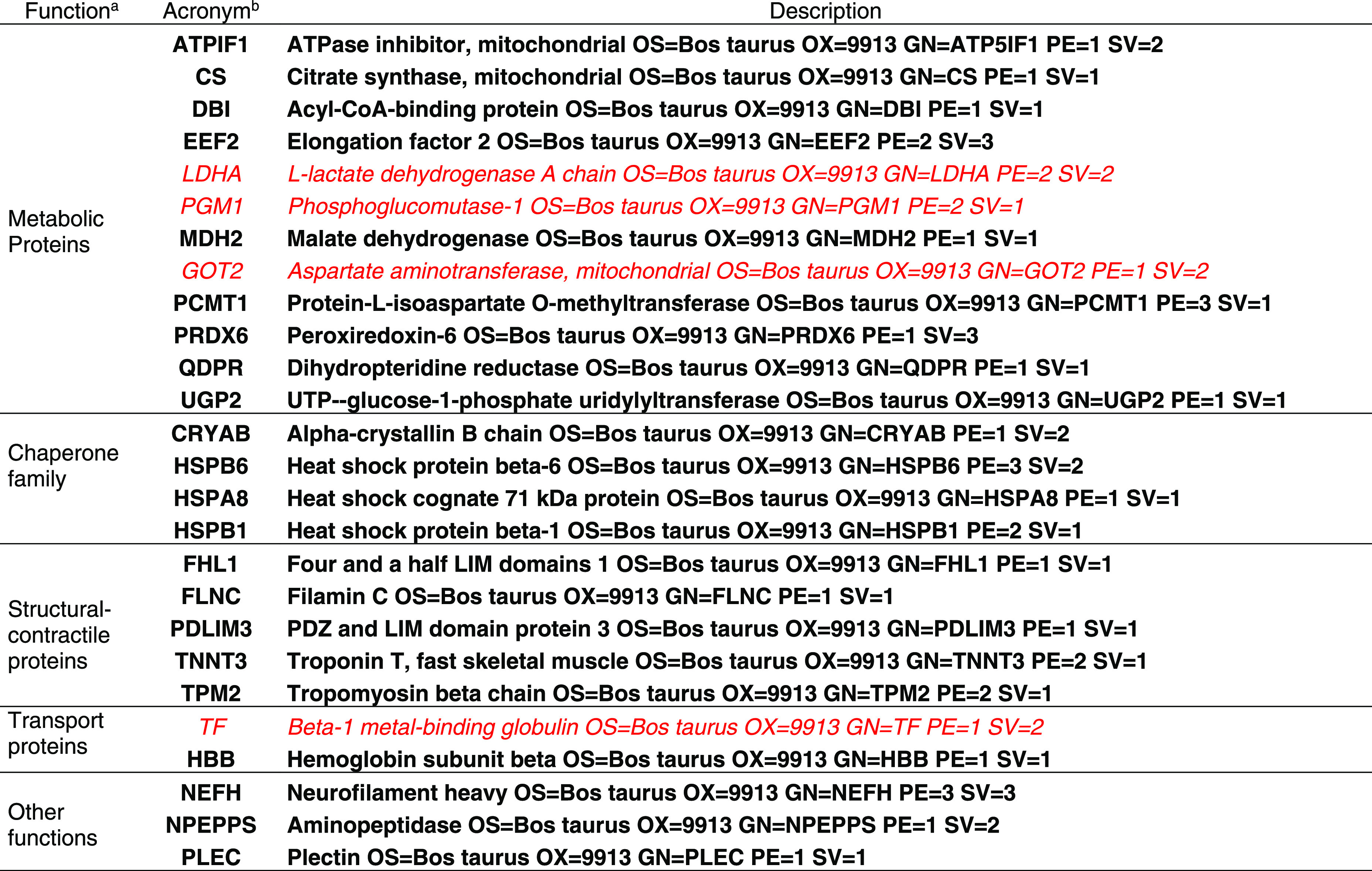
Protein Biomarkers Characterizing Normal (Cursive Red) and High (Bold Black) pHu Pooled Meat Sample Groups Assayed

a Biological functions are detailed in the main text (see the Biological Function of Proposed Biomarkers section).

b Identification details are originally from Table S2. All descriptors found in both meat groups were assayed at different significance levels with the exception of DBI that was exclusive for high pHu meat (Table S4).

### Biological Functions of Proposed Biomarkers

To facilitate the understanding of results, a discussion about the functional analysis of protein biomarkers is first overviewed and then discussed in detail in different subsections.

**Figure 1:**
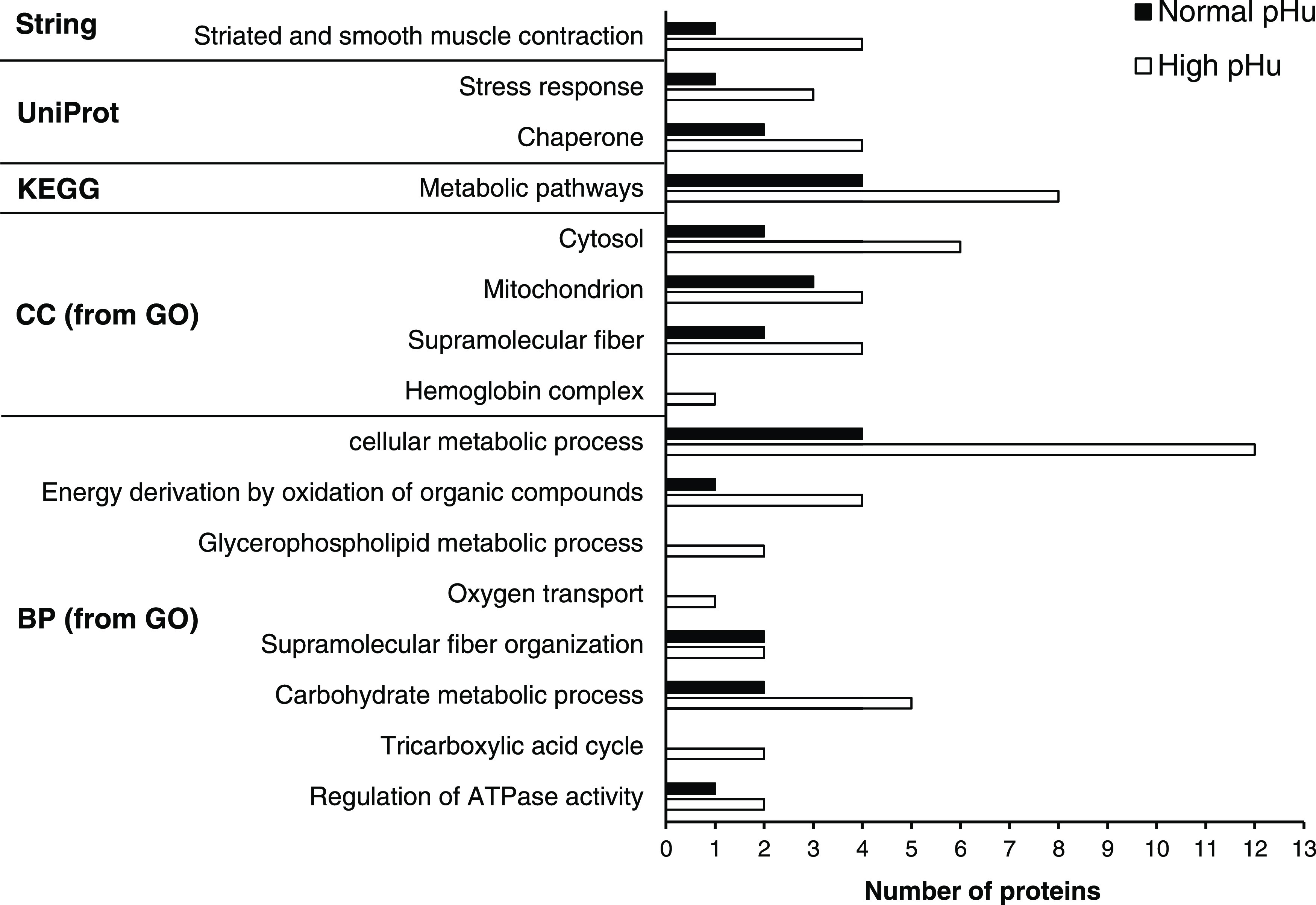
Classification of the proposed protein biomarkers (Table 1) from normal (■) and high (□) pHu meat samples considering Gene Ontology (GO) terms, KEGG pathways, annotated keywords (UniProt), and local network cluster (STRING software). GO terms: BP, biological process; CC, cellular component.

**Figure 2:**
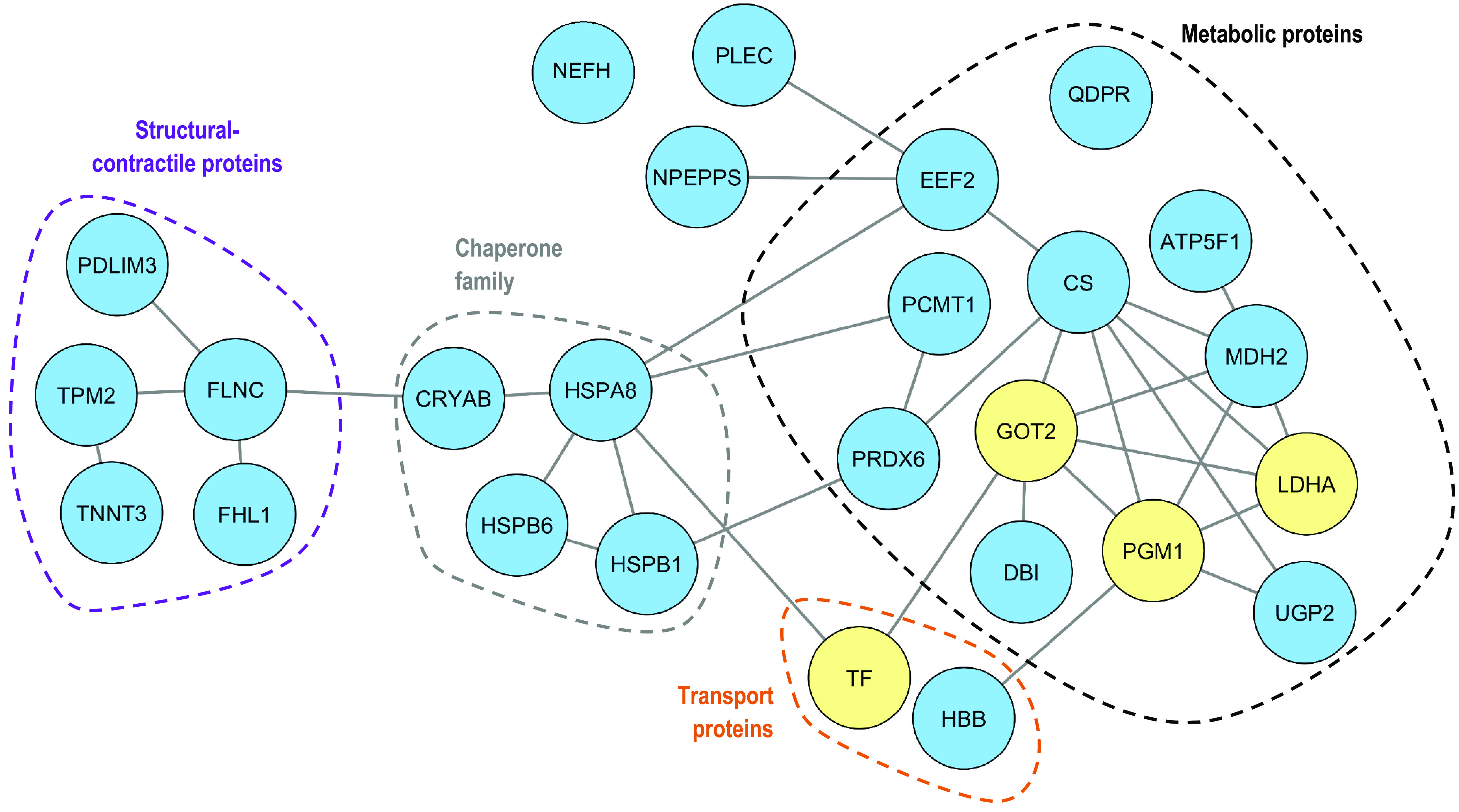
Cytoscape protein–protein interaction network of the proposed protein biomarkers (Table 1) from normal and high pHu meat groups studied. Network nodes (circles) represent proteins and lines denote protein–protein functional associations (threshold: >0.4 medium confidence level). Yellow and blue colors represent upregulated proteins found in normal and high pHu samples, respectively. Dashed lines delimit protein clustering according to the functional role.

The functional analysis of the 26 protein biomarkers listed in Table 1 evidenced their participation in different biological processes, as depicted in Figure 1. Proteins belong to different locations such as extracellular space, cell membrane, and inside cells as a constituent component of the cytoplasm, cytosol, or mitochondrion (Figure 1). The interaction network of proteins (Figure 2 from Cytoscape analysis with yellow and blue colors for characteristic normal and high pHu proteins, respectively) and interaction strength among them (Figure S5 from STRING analysis) revealed several remarkable facts. All proteins were clustered into a single network with the only exception of NEFH and QDPR. Furthermore, proteins were mainly grouped according to biological functions, as shown in Figure 2: metabolic proteins (ATPIF1, CS, DBI, EEF2, GOT2, MDH2, LDHA, PCMT1, PGM1, PRDX6, QDPR, and UGP2), proteins belonging to the chaperone family (CRYAB, HSPA8, HSPB1, and HSPB6), structural-contractile proteins (FHL1, FLNC, PDLIM3, TNNT3, and TPM2), and transport functions (HBB and TF). Only NEFH, NPEPPS, and PLEC were outsiders regarding these four principal functionalities and were classified as “others”. Metabolic proteins had the largest number of interacting partners and evidenced their key role in the response mechanism induced by PSS. Moreover, the cluster conformed by chaperones connected metabolic enzymes with structural-contractile proteins. This finding can be explained considering the participation of chaperones in different protein configuration processes (assembly/disassembly, folding/unfolding, translocation, and actin organization) and their interaction with damaged proteins under stressed conditions.^21^

### Metabolic Proteins

This group is composed of 12 metabolic enzymes that in general are directly or indirectly related to carbohydrate metabolism (CS, LDHA, MDH2, PGM1, and UGP2), tricarboxylic acid (TCA) cycle (CS and MDH2), lipids (DBI and PRDX6), amino acids (GOT2 and QDPR), catabolism/degradation, and other metabolic processes (ATPIF1, EEF2, and PCMT1). Among them, only GOT2, LDHA, and PGM1 were overabundant in the normal pHu group (Table 1).

It has been widely described that the PSS response is associated with the depletion of muscle glycogen stores prior to slaughter, causing a reduction in the substrate availability of anaerobic glycolysis postmortem.^22^ This perturbs glycolytic potential, which is a measurement of the remaining amount of glycogen and lactate in the muscle, affecting the metabolism of key enzymes involved in this pathway.^22−24^ Although the association between pre-slaughter stress and muscle glycogen depletion has been extensively studied in ruminants, the linkage between PSS and the postmortem glycolytic rate remains unclear.^25^ Regarding CS, which catalyzes the initial reaction of the TCA cycle performing the irreversible condensation of acetyl (CoA) with oxalacetate to form citrate, there are no studies reporting any relationship with high pHu meats. However, some studies demonstrated the overabundance of some TCA metabolites such as citric acid in high pHu meats,^26^ explaining the upregulation of this enzyme in our study.

MDH2, which is involved in glucose production^27^ when glycogen is not available, was characteristic of the high pHu sample (Table 1) as previously reported.^18^ In line with this, some authors also observed higher levels of UGP2 in high pHu meats. This could be due to the enhanced gluconeogenesis required for replenishing the low glycogen levels by promoting the flux of glucose toward glycogen synthase.^17^ UGP2 participates in the biosynthesis of glycogen by transferring a glucose moiety from glucose 1-phosphate to MgUTP, giving rise to the formation of UDP-glucose and MgPPi. There are, however, other authors that reported a lower abundance of UGP2 in high pHu meat samples.^5^

PGM1 and LDHA were found as overabundant proteins in the normal group. Previous studies evidenced the downregulation of PGM1 in high pHu meats,^9,17^ suggesting that this could be related to glycogen depletion before slaughter since PSS notably reduces the metabolism of this enzyme. In this line, Fuente-García et al. claimed that this fact might depend on its phosphorylated form.^9^ Previous results stated that PGM1 underwent phosphorylation changes between high and normal pHu meats,^10^ suggesting that the phosphorylation state may alter the rate of conversion of glucose 1-phosphate to glucose 6-phosphate, inducing differences in the rate of pH decline.^28^ Concerning LDHA, which catalyzes the reversible conversion of pyruvate to lactate, there were reports on its preponderance in normal pH meats,^18^ confirming the results achieved in current research. However, other authors found decreased levels of LDHA in normal pHu muscle extracts, suggesting that this might be due to muscle physiology and not pHu variations.^8^ The literature also reported that increased LDHB concentrations could be associated with an accelerated postmortem pH decline.^29^ Taking into account that PSS animals have limited glucose reserves, increased energy demands caused by the PSS response may alter carbohydrate metabolism, thus enhancing the activity of those enzymes that are directly related to ATP production. It was demonstrated how carcasses yielding abnormal dark cutting meat (pHu ≤ 5.8) might have reduced glycolysis rates at early postmortem times, giving rise to low concentrations of energy-related proteins.^8^ Therefore, further research is needed to better understand the role that each enzyme plays in postmortem muscle metabolism.

Outside their key role in carbohydrate metabolism, CS and MDH2 (overabundant in the high meat group) are also involved in the TCA cycle by catalyzing the initial reaction of the cycle and the oxidation of malate to oxaloacetate, respectively. The TCA cycle is the final common pathway for the oxidation of fuel molecules (i.e., amino acids, fatty acids, and carbohydrates) associated with the production of energy and reduction equivalents (NADH and FADH_2_) participating in mitochondrial electron transfer. Since PSS animals have less glycogen stores prior to slaughter, muscle cells would need alternative energy sources such as the TCA cycle for restoring ATP levels, explaining the overabundance of CS and MDH2 in the high pHu sample group. Although alternative energy pathways would be preferably activated before slaughter, some authors reported that mitochondria can still consume oxygen in postmortem muscle even after 60 days of storage under vacuum packaging.^30^ This suggests that oxidative metabolism (i.e., TCA cycle and/or oxidative phosphorylation) might be also activated in postmortem muscle to maintain cell homeostasis.^17,18^ In dark cutting beef, higher NADH levels lead to higher oxygen consumption and influence the myoglobin redox state,^17,30^ affecting the meat color. NADH and other substrates such as malate can also limit available oxygen to myoglobin, promoting the formation of deoxymyoglobin,^26^ giving rise to a darker color, and limiting brown or metmyoglobin (MetMb) formation. In this line, several studies stated that a higher muscle pHu can increase the activity of several enzymes involved in MetMb, reducing the activity and oxygen consumption.^30,31^

Fatty acid metabolism, especially fatty acid β-oxidation, is an important pathway of energy metabolism. In this study, DBI and PRDX6 were characteristic of the high pHu meat group (Table 1). Although these proteins have not been previously related to high pHu meats, it was observed that other enzymes involved in fatty acid metabolism were upregulated in dark cutting meats.^18^ Again, this clearly suggests that high pHu meats probably exploit alternative metabolism pathways such as lipid oxidation to obtain energy from oxidative phosphorylation, improving both mitochondrial oxygen consumption and mitochondrial respiration.^17,18,32^ Apart from its role in fatty acid metabolism, PRDX6 is an antioxidant enzyme that contributes to the detoxification of reactive oxygen species and it was proposed as a potential indicator of oxidative stress.^33^

Regarding amino acid degradation as another alternative source of energy when other substrates are limited, QDPR, which is an essential enzyme for phenylalanine and tyrosine degradation, was characteristic of the high pHu meat group. On the contrary, GOT2, which participates in aspartate metabolism through the reversible transamination of aspartate and 2-oxoglutarate to form oxaloacetate and glutamate, was representative of the normal pHu sample group. Although some authors directly linked GOT2 to high pHu meats,^18^ it also takes part in different biological processes, deserving further research to understand its regulation in meat depending on pHu.

The remaining metabolic proteins ATPIF1, EEF2, and PCMT1 were upregulated in the high pHu meat group. EEF2 catalyzes the GTP-dependent ribosomal translocation step during translation elongation, playing an important role in protein synthesis. Some studies reported that inhibition of EEF2 and eukaryotic translation initiation factor 2 (EIF2) activity by phosphorylation might occur as a response to cellular stress, contributing to the suppression of protein synthesis during exercise/contractile activity.^34−36^ Similarly, other results suggested that EIF2, also involved in protein synthesis through the initiation step of RNA translation, showed higher abundance in high pHu meats.^18^ Since the occurrence of high pHu meats is intimately linked to the animal stress condition, this can explain increased EEF2 phosphorylation in the high pHu sample that inhibited skeletal muscle protein synthesis. Other proteins not previously described as biomarkers of high pHu meats were ATPIF1 and PCMT1. The former is an enzyme that negatively regulates ATPase activity, reducing the rate of ATP hydrolysis when the potential of the mitochondrial membrane falls, explaining its overabundance in the high pH sample group. Second, PCMT1 catalyzes the methyl esterification of l-isoaspartyl and d-aspartyl residues in peptides and proteins resulting from the spontaneous decomposition of normal l-aspartyl and l-asparaginyl residues. It plays an essential role in the repair and/or degradation of damaged proteins, especially in methionine degradation, then explaining its upregulation in the high pHu sample.

### Chaperone and Stress-Related Proteins

Proteins CRYAB, HSPA, HSPB6, and HSPB1 belonging to the chaperone family were displayed in the core of the network (Figure 2) and characterized the high pHu meat group in accordance with previous results.^9,18^ These proteins were commonly studied by their role in the stress response, actin stability, and apoptotic signaling pathways. Under stressful conditions, they play a major part as essential molecular chaperones interacting with damaged proteins to preserve their function.^37,38^ Considering the protective activity of chaperones, it is logical to assume that PSS animals would have higher levels of HSPs to maintain cell integrity and to prevent the activation of apoptosis signaling pathways. Triggering of this process depends on the nature of the initial stimulus, finding animal stress as one of the most relevant factors where the involvement of HSPs has been described as antiapoptotic players counteracting the caspase activity.^37,38^ Particular functionalities of HSPs seem to be linked to their phosphorylation status as demonstrated by Mato et al.,^10^ finding noticeable differences in HSPB1 and HSPB6 from normal and high pHu meats. Therefore, additional research is needed to completely understand the specific role of HSPs according to post-translational modifications such as phosphorylation.

### Structural-Contractile Proteins

During the conversion of muscle into meat, there are complex interactions between biochemical processes that influence the final meat texture characteristics such as fragmentation of myofibrils. Proteolytic degradation of several structural proteins (i.e., titin, nebulin, troponin T, desmin, filamin, and vinculin) plays a major task in the development of meat tenderness.^39−41^ It is noteworthy that proteins involved in muscle contraction are insoluble and should be represented in the myofibrillar subproteome. However, high pHu meats favor their solubilization^24^ and facilitate their extraction within the sarcoplasmic fraction.^9^ This can explain the main occurrence of FHL1, FLNC, PDLIM3, TNNT3, and TPM2 in the high meat group assayed (Table 1). However, other studies pointed out that FLNC, which is a protein that cross-links actin cytoskeleton filaments into a dynamic structure,^42^ was overregulated in the normal pHu sample group.^5^ TPM2 binds to actin filaments in muscle cells and plays a central role, in association with TNNT3, in the calcium-dependent regulation of vertebrate striated muscle contraction. Participation of TPM2 and TNNT3 in PSS muscle is quite controversial since both proteins were found up- and downregulated in both normal and high pHu meats. While Mato et al.^10^ found higher TNNT3 phosphorylation levels in high pHu samples, other authors reported that tropomyosin α-1 chain levels were lower in high pHu meats.^8^ In this line, some authors studied the influence of pHu in muscle contraction, finding that high pHu meats prevented protein denaturation, reducing muscle transverse shrinkage and increasing WHC, thus contributing to dark color.^43^ These findings could explain the upregulation of TPM2 and TNNT3 in the high pHu sample assayed (Table 1).

This study describes, for the first time, the presence of proteins FHL1 and PDLIM3 in relation to high pHu meat characteristics. While PDLIM3 may be relevant in the organization of actin filament arrays within muscle cells, FHL1 is involved in the regulation of muscle development. Despite these findings, further research is needed to refine the understanding of the influence of these proteins in the occurrence of high pHu meats.

### Transport Proteins

HBB is a heterotetrameric oxygen transport protein found in red blood cells and involved in oxygen transport from the lungs to various peripheral tissues. It was previously reported that respiration machinery has an enhanced functionality in high pHu muscles.^17,18,24,26^ As a result, increased oxygen consumption would favor its transportation by specific proteins such as HBB, explaining its overabundance in the high pHu sample (Table 1). In contrast, the iron transportation TF protein was less abundant in this assayed meat group. Although TF is necessary for hemo-based protein biosynthesis, previous studies reported that TF levels decreased in the case of inflammation,^44^ as occurs under stress situations. In any case, further research is needed to clarify the role of transport proteins on the apparition of defective meat.

### Others

Other proteins not assigned to any of the aforementioned biological functions that were found as descriptors of the high pHu meat group were NEFH, NPEPPS, and PLEC (Table 1). A higher abundance of NPEPPS, an essential aminopeptidase for peptide catabolism, might be a sign of a greater amino acid degradation as an alternative energy source due to the lack of carbohydrate energy supply. Previous studies described the overabundance of some amino acid metabolic enzymes^18^ and reduced amino acid concentrations in high pHu meats.^32^ An increased pHu in meat favors the solubilization of myofibrillar contractile proteins such as PLEC,^9,24^ explaining its overabundance in the high pHu sample. However, upregulation of NEFH, involved in DNA binding, axon development, and nucleosome assembly, is still unclear and deserves further investigation.

Overall, the results obtained in this study suggest that different alternative energy sources could be activated in PSS animals as a consequence of the reduced glycolytic metabolism. This would indicate that cellular energy arises not only from muscle glycogen but also other compounds could contribute to ATP production in animals, yielding high pHu meats by the activation of other biochemical pathways such as lipid and amino acid metabolism/degradation, TCA cycle, and oxidative phosphorylation. This also suggests that the greater oxidative stress and ROS production in PSS animals could lead to an early onset of apoptosis, increasing the upregulation of some antiapoptotic proteins such HSPs in high pHu meats. However, further research is needed to understand whether these energy pathways (i.e., TCA cycle and/or oxidative phosphorylation) would still remain active in postmortem muscle. Additionally, structural-contractile proteins seemed to be differentially regulated between high and normal pHu meats, being the detailed explanation of this a matter of further investigations.

The efficiency, rapidity, and simplicity of the proteomic approach proposed in this work gave rise to clear results dealing with main biochemical pathways underlying the occurrence of high pHu meats. Rigorous selection of protein candidates yielded 26 meat biomarkers that clearly characterized meat groups assayed. Label-free relative MS^1^ peptide quantification analysis demonstrated the usefulness of the coarse protein score ratio indicator as an interesting strategy for the rapid screening of potential protein biomarkers. The functional analysis of the proposed discriminant proteins allowed their clustering according to four main biological functions, namely, metabolic proteins, chaperone and stress-related proteins, structural-contractile proteins, and transport proteins. Interestingly, this straightforward proteomic strategy first described some biomarkers associated with high pHu meats.

The results achieved can promote the implementation of the proposed methodology to create new insights addressing the rapid assessment of meat quality.

## Supporting Information Available

Highlights (PDF)
In-house library used for label-free peptide quantification (Table S1) (XLS)
Identified proteins from normal (cursive red) and high (bold black) pHu pooled meat sample groups assayed (Table S2) (XLS)
Specific protein descriptors found in normal (cursive red) and high (bold black) pHu pooled meat sample groups approached by the preliminary protein score strategy. Proteins shared by NORMAL and HIGH pHu pooled meat sample groups assayed with a minimum individual protein score ratio (fold-change) of 2 (Table S3B) (Table S3A) (XLSX)
Label-free MS1 relative quantification of proteotypic peptides from potential protein biomarkers proposed by the preliminary protein score approach (Table S4) (XLSX)
Peptide score distribution achieved by normal (Figure S1A) and high (Figure S1B) pHu meat samples assayed to determine the individual ion score threshold indicating identity or extensive homology (*P* < 0.05) (PDF)
Protein sequence coverage and peptide mass dispersion reports of identified proteins from normal (GAPDH, Figure S2A and BIN1, Figure S2B) and high (HSPA8, Figure S2C and DBI, Figure S2D) pHu meat samples assayed. The nomenclature of protein families shown is from Table S2 (PDF)
MS/MS univocal amino acid sequencing of tryptic peptides from proteins detailed in Figure S3 found in normal (GAPDH, Figure S3A and BIN1, Figure S3B) and high (HSPA8, Figure S3C and DBI, Figure S3D) pHu meat samples assayed. The nomenclature of protein families shown is from Table S2 (PDF)
MS/MS interrogation of a tryptic peptide from the PYGM protein found in the normal pHu sample with several feasible amino acid sequences with one clearly distinguished. The nomenclature of the protein studied is from Table S2 (Figure S4) (PDF)
STRING protein–protein interaction network of the proposed protein biomarkers (Table 1) from normal and high pHu meat samples. Network nodes (circles) represent proteins, lines denote protein–protein functional associations, and line thickness indicates the association strength (threshold: >0.4 medium confidence level) (Figure S5)(PDF)
Samples were studied by merging full MS1 and data-dependent MS/MS (dd-MS^2^) analyses (PDF)
