# Supplementary material for: Protein Biomarkers of Bovine Defective Meats at a Glance: Gel-Free Hybrid Quadrupole-Orbitrap Analysis for Rapid Screening
Source: J Agric Food Chem. 2021 Jun 25;69(26):7478–87. doi: 10.1021/acs.jafc.1c02016 (PMC8278482; doi:10.1021/acs.jafc.1c02016)
Supplement: Supplementary file 1 — jf1c02016_si_001.pdf [file jf1c02016_si_001.pdf]

Highlights

-Proteomic profiles of normal and high pHu meats were assessed by LC-HRMS analysis.

-A straightforward study of direct protein extracts eased characterization of samples.

-Meat groups assayed were characterized through 26 protein biomarkers.

-Metabolic, chaperone, contractility and transport were main functions of descriptors.

-This efficient and rapid methodology can be readily implemented in meat research.
