# Supplementary material for: Protein Biomarkers of Bovine Defective Meats at a Glance: Gel-Free Hybrid Quadrupole-Orbitrap Analysis for Rapid Screening
Source: J Agric Food Chem. 2021 Jun 25;69(26):7478–87. doi: 10.1021/acs.jafc.1c02016 (PMC8278482; doi:10.1021/acs.jafc.1c02016)
Supplement: Supplementary file 6 — jf1c02016_si_006.pdf [file jf1c02016_si_006.pdf]

Figure S1A

MATRIX SCIENCE MASCOT Search Results

Search title : Conversion of QexNORMALhesiOFF.mzXML to mascot generic
MS data file : QexNORMALhesiOFF.mgf
Database : UP9136_B_taurus 20201007 (37,880 sequences; 22,676,167 residues)
Timestamp : 1 Dec 2020 at 12:24:06 GMT

Re-search ◉ All ○ Non-significant ○ Unassigned [help] Export As XML

Not what you expected? Try the select summary.

▸ **Search parameters**

▾ ***Score distribution***

NORMAL

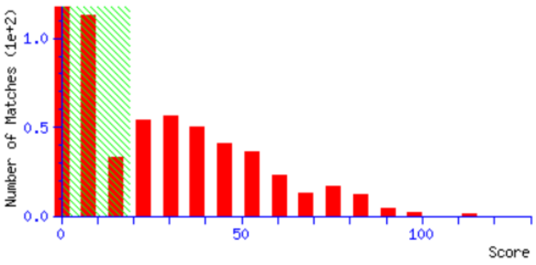

Peptide score distribution. Ions score is $-10\log(P)$, where P is the probability that the observed match is a random event.
There are **303** peptide matches above identity threshold and **314** matches above homology threshold for **10,779** queries. On average, individual ions scores **> 19** (beyond green shading) indicate **identity or extensive homology** (p<0.05).

Figure S1B

MATRIX SCIENCE MASCOT Search Results

Search title : Conversion of QexDFDhesiOFF.mzXML to mascot generic
MS data file : QexDFDhesiOFF.mgf
Database : UP9136_B_taurus 20201007 (37,880 sequences; 22,676,167 residues)
Timestamp : 1 Dec 2020 at 12:13:03 GMT

Re-search ◉ All ○ Non-significant ○ Unassigned [help] Export As XML

Not what you expected? Try the select summary.

▸ **Search parameters**

▾ ***Score distribution***

HIGH pHu

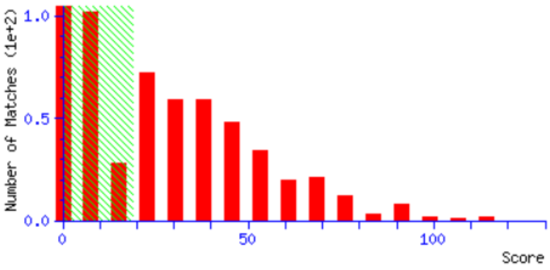

Peptide score distribution. Ions score is $-10\log(P)$, where P is the probability that the observed match is a random event.
There are **329** peptide matches above identity threshold and **339** matches above homology threshold for **10,859** queries. On average, individual ions scores **> 19** (beyond green shading) indicate **identity or extensive homology** (p<0.05).
