# Supplementary material for: Protein Biomarkers of Bovine Defective Meats at a Glance: Gel-Free Hybrid Quadrupole-Orbitrap Analysis for Rapid Screening
Source: J Agric Food Chem. 2021 Jun 25;69(26):7478–87. doi: 10.1021/acs.jafc.1c02016 (PMC8278482; doi:10.1021/acs.jafc.1c02016)

Figure S2A

MASCOT Search Results

Protein View: P10096

Glyceraldehyde-3-phosphate dehydrogenase OS=Bos taurus OX=9913 GN=GAPDH PE=1 SV=4

**Database:**	UP9136_B_taurus
**Score:**	523
**Monoisotopic mass (M_r):**	35845
**Calculated pI:**	8.50

Sequence similarity is available as an NCBI BLAST search of P10096 against nr.

Search parameters

**MS data file:**	QexNORMALhesiOFF.mgf
**Enzyme:**	Trypsin: cuts C-term side of KR unless next residue is P.

Protein sequence coverage: 46%

Matched peptides shown in ***bold red***.

1 M**VKVGVNGFG** **R**IGRLVTRAA FNSGKVDIVA INDPFIDLHY MVYMFQYDST
51 HGKFNGTVKA ENGKLVINGK **AITIFQERDP** **ANIKWGDAGA** **EYVVESTGVF**
101 TTMEKAGAHL KGGAKRVIIS APSA**DAPMFV** **MGVNHEK**YNN TLK**IVSNASC**
151 **TTNCLAPLAK** VIHDHFGIVE GLMTTVHAIT ATQK**TVDGPS** **GK**LWRDGR**GA**
201 **AQNIIPASTG** **AAK**AVGK**VIP** **ELNGKLTGMA** **FRVPTPNVSV** **VDLTCR**LEKP
251 AKYDEIKKVV K**QASEGPLK**G ILGYTEDQVV SCDFNSDTHS STFDAGAGIA
301 LNDHFVK**LIS** **WYDNEFGYSN** **RVVDLMVHMA** **SKE**

Unformatted sequence string: 333 residues (for pasting into other applications).

Sort by ◉ residue number ○ increasing mass ○ decreasing mass
Show ◉ matched peptides only ○ predicted peptides also

Query	Start – End	Observed	Mr(expt)	Mr(calc)	ppm	M	Score	Expect	Rank	U	Peptide
4493	2 – 11	516.8004	1031.5863	1031.5876	-1.31	1	33	0.00087	1	U	M.VKVGVNGFGR.I
2533	4 – 11	805.4307	804.4234	804.4243	-1.08	0	30	0.0015	1	U	K.VGVNGFGR.I
4107	71 – 78	489.2738	976.5331	976.5342	-1.07	0	38	0.00028	1	U	K.AITIFQER.D
4108	71 – 78	977.5408	976.5335	976.5342	-0.69	0	32	0.0029	1	U	K.AITIFQER.D
8170	71 – 84	808.4429	1614.8712	1614.8729	-1.07	1	51	1.8e-05	1	U	K.AITIFQERDPANIK.W
7019	73 – 84	716.3823	1430.7501	1430.7518	-1.16	1	27		1	U	I.TIFQERDPANIK.W
6250	74 – 84	665.8590	1329.7035	1329.7041	-0.45	1	34		1	U	T.IFQERDPANIK.W
8675	85 – 100	843.8867	1685.7589	1685.7573	0.92	0	26		1	U	K.WGDAGAEYVVESTGVF.T
7266	125 – 137	737.8442	1473.6739	1473.6745	-0.36	0	49		1	U	A.DAPMFVMGVNHEK.Y
8817	144 – 160	852.4260	1702.8375	1702.8382	-0.42	0	50		1	U	K.IVSNASCTTNCLAPLAK.V + [-2.0156 at T9]
8832	144 – 160	853.4337	1704.8528	1704.8539	-0.64	0	49	2.3e-05	1	U	K.IVSNASCTTNCLAPLAK.V
8843	144 – 160	853.9348	1705.8551	1705.8509	2.45	0	34		1	U	K.IVSNASCTTNCLAPLAK.V + [+0.9970 at T9]
8847	144 – 160	854.4355	1706.8564	1706.8581	-0.98	0	30		1	U	K.IVSNASCTTNCLAPLAK.V + [+2.0042 at T9]
2194	185 – 192	760.3824	759.3751	759.3763	-1.56	0	68	6.1e-07	3	U	K.TVDGPSGK.L
3054	199 – 207	854.4725	853.4652	853.4657	-0.64	0	26		1	U	R.GAAQNIIPA.S
6623	199 – 213	685.3741	1368.7336	1368.7361	-1.81	0	68	7.2e-07	1	U	R.GAAQNIIPASTGAAK.A
6624	199 – 213	1369.7434	1368.7361	1368.7361	0.024	0	77	5.6e-08	1	U	R.GAAQNIIPASTGAAK.A
1096	207 – 213	605.3241	604.3168	604.3180	-1.99	0	27		1	U	P.ASTGAAK.A
3208	218 – 225	869.5096	868.5024	868.5018	0.65	0	48	6.8e-05	1	U	K.VIPELNGK.L
3218	218 – 225	870.4901	869.4828	869.4858	-3.49	0	40		1	U	K.VIPELNGK.L + [+0.9840 at N6]
2449	226 – 232	795.4181	794.4108	794.4109	-0.093	0	27	0.0021	1	U	K.LTGMAFR.V
7407	233 – 246	750.3981	1498.7817	1498.7814	0.22	0	63	1.2e-06	1	U	R.VPTPNVSVVDLTCR.L
2609	262 – 269	812.4147	811.4075	811.4076	-0.15	0	35		1	U	K.QASEGPLK.G + [-17.0265 at N-term]
2784	262 – 269	829.4406	828.4333	828.4341	-0.94	0	60	6.3e-06	1	U	K.QASEGPLK.G
9241	308 – 321	882.4061	1762.7977	1762.7951	1.49	0	58	4.7e-06	1	U	K.LISWYDNEFGYSNR.V
6502	322 – 333	679.8436	1357.6726	1357.6734	-0.59	1	42	0.00032	1	U	R.VVDLMVHMASKE.-
6503	322 – 333	679.8455	1357.6765	1357.6734	2.28	1	31	0.0012	1	U	R.VVDLMVHMASKE.-
5772	323 – 333	630.3094	1258.6043	1258.6050	-0.49	1	33		1	U	V.VDLMVHMASKE.-
5209	324 – 333	580.7749	1159.5353	1159.5365	-1.12	1	28		1	U	V.DLMVHMASKE.-

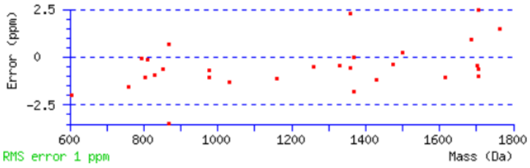

Figure S2B

MATRIX SCIENCE MASCOT Search Results

Protein View: A0A3Q1LV21

Bridging integrator 1 OS=Bos taurus OX=9913 GN=BIN1 PE=4 SV=1

**Database:**	UP9136_B_taurus
**Score:**	22
**Monoisotopic mass (M_r):**	66196
**Calculated pI:**	5.32

Sequence similarity is available as an NCBI BLAST search of A0A3Q1LV21 against nr.

Search parameters

**MS data file:**	QexNORMALhesiOFF.mgf
**Enzyme:**	Trypsin: cuts C-term side of KR unless next residue is P.

Protein sequence coverage: 1%

Matched peptides shown in ***bold red***.

```
  1 MAEMGSKGVT AGKIASNVQK KLTRAQEKVL QKLGKADETK DEQFEQCVQN
 51 FNKQLTEGTR LQKDLRTYLA SVKAMHEASK KLNECLQEVY EPDWPGRDEA
101 SKIAENNDLL WMDYHQKLVD QALLTMDTYL GQFPDIKSRI AKRGRKLVDY
151 DSARHHYESL QTAKKKDEAK IAKPVSLLEK AAPQWCQGKL QAHLVAQTNL
201 LRNQAEEELI KAQKVFEEMN VDLQEELPSL WNSRVGFYVN TFQSIAGLEE
251 NFHKEMSKLN QNLNDVLISL EKQHGSNTFT VKAQPRKKTK LFSRLRRKKN
301 SDNVPAKGNK SPSPPPDGSP AATPEIRVNH EPEPAGAATP GATLPKSPSQ
351 LRKGPPVPPP PKHTPSKEVK QEQILSLFDD TFVPEISVTT PSQFEAPGPF
401 SEQASLLDLD FDPLPPVASP VKAPTPSGQS IPWDLWEPTE SPAGSLPSGE
451 PSAAEGTFAV AWPSQTAEPG PAQPAEASEA AGAQEPGETT TSEAASSSLP
501 AVVVETFSAT VNGTVESGSG AGRLDLPPGF MFKVQAQHDY TATDTDELQL
551 KAGDVVLVIP FQNPEEQDEG WLMGVKESDW NQHKELEKCR GVFPENFTER
601 VQ
```

Unformatted sequence string: 602 residues (for pasting into other applications).

Sort by ◉ residue number ○ increasing mass ○ decreasing mass
Show ◉ matched peptides only ○ predicted peptides also

Query	Start – End	Observed	Mr(expt)	Mr(calc)	ppm	M	Score	Expect	Rank	U	Peptide
5373	591 – 600	598.2905	1194.5665	1194.5669	-0.37	0	22	0.031	1	U	R.GVFPENFTER.V

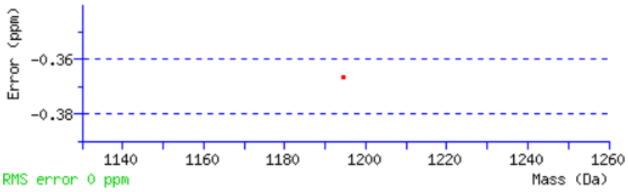

Figure S2C

MATRIX SCIENCE MASCOT Search Results

Protein View: A0A3Q1LMS5

Heat shock cognate 71 kDa protein OS=Bos taurus OX=9913 GN=HSPA8 PE=1 SV=1

**Database:**	UP9136_B_taurus
**Score:**	315
**Monoisotopic mass (M_r):**	71720
**Calculated pI:**	5.37

Sequence similarity is available as an NCBI BLAST search of A0A3Q1LMS5 against nr.

Search parameters

**MS data file:**	QexDFDhesiOFF.mgf
**Enzyme:**	Trypsin: cuts C-term side of KR unless next residue is P.

Protein sequence coverage: 10%

Matched peptides shown in ***bold red***.

1 MSKGPAVGID LGTTYSCVGV FQHGKVEIIA NDQGNR**TTPS** **YVAFTDTER**L
51 IGDAAKNQVA MNPTNTVFDA KRLIGRRFDD AVVQSDMKHW PFMVVNDAGR
101 PKVQVEYKGE TKSFYPEEVS SMVLTKMKEI AEAYLGKVST IVCIFAYPTL
151 PAYFNDSQRQ ATKDAGTIAG LNVLR**IINEP** **TAAAIAYGLD** **KK**VGAERNVL
201 IFDLGGGTFD VSILTIEDGI FEVKSTAGDT HLGGEDFDNR MVNHFIAEFK
251 RKHKKDISEN KRAVRRLRTA CERAKRTLSS STQASIEIDS LYEGIDFYTS
301 ITR**ARFEELN** **ADLFR**GTLDP VEKALRDAKL DKSQIHDIVL VGGSTRIPKI
351 QK**LLQDFFNG** **KELNK**SINPD EAVAYGAAVQ AAILSGDKSE NVQDLLLLDV
401 TPLSLGIETA GGVMTVLIKR NTTIPTKQTQ TFTTYSDNQP GVLIQVYEGE
451 RAMTKDNNLL GKFELTGIPP APRGVPQIEV TFDIDANGIL NVSAVDKSTG
501 KENKITITND KGRLSKEDIE RMVQEAEKYK AEDEKQRDKV SSK**NSLESYA**
551 **FNMK**ATVEDE KLQGKINDED KQKILDKCNE IINWLDKNQT AEKEEFEHQQ
601 KELEKVCNPI ITKLYQSAGG MPGGMPGGMP GGFPGGGAPP SGGASSGPTI
651 EEVD

Unformatted sequence string: 654 residues (for pasting into other applications).

Sort by ◉ residue number ○ increasing mass ○ decreasing mass
Show ◉ matched peptides only ○ predicted peptides also

Query	Start – End	Observed	Mr(expt)	Mr(calc)	ppm	M	Score	Expect	Rank	U	Peptide
7301	37 – 49	744.3530	1486.6914	1486.6940	-1.76	0	76	1.1e-07	1		R.TTPSYVAFTDTER.L
9431	176 – 192	596.6680	1786.9823	1786.9828	-0.33	1	71	2.6e-07	1	U	R.IINEPTAAAIAYGLDKK.V
9432	176 – 192	894.4993	1786.9841	1786.9828	0.70	1	54	1.3e-05	1	U	R.IINEPTAAAIAYGLDKK.V
7255	304 – 315	740.8813	1479.7480	1479.7470	0.69	1	47	4.3e-05	1	U	R.ARFEELNADLFR.G
7256	304 – 315	494.2567	1479.7482	1479.7470	0.83	1	38	0.00027	1	U	R.ARFEELNADLFR.G
7824	353 – 365	522.6164	1564.8274	1564.8249	1.55	1	41	0.00015	1	U	K.LLQDFFNGKELNK.S
7825	353 – 365	783.4212	1564.8279	1564.8249	1.88	1	47	3.7e-05	1	U	K.LLQDFFNGKELNK.S
6124	544 – 554	652.3024	1302.5902	1302.5914	-0.93	0	57	6.8e-06	1	U	K.NSLESYAFNMK.A
6141	544 – 554	652.8045	1303.5945	1303.5884	4.61	0	57		1	U	K.NSLESYAFNMK.A + [+0.9970 at A7]

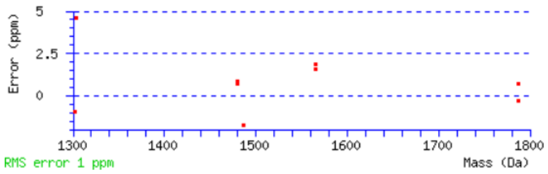

Figure S2D

MASCOT Search Results

Protein View: A0A3Q1M8I0

Acyl-CoA-binding protein OS=Bos taurus OX=9913 GN=DBI PE=1 SV=1

**Database:**	UP9136_B_taurus
**Score:**	43
**Monoisotopic mass (M_r):**	10736
**Calculated pI:**	6.28

Sequence similarity is available as an NCBI BLAST search of A0A3Q1M8I0 against nr.

Search parameters

**MS data file:**	QexDFDhesiOFF.mgf
**Enzyme:**	Trypsin: cuts C-term side of KR unless next residue is P.

Protein sequence coverage: 17%

Matched peptides shown in ***bold red***.

1 MSQAEFDKAA EEVKHLK**TKP** **ADEEMLFIYS** **HYK**QATVGDI NTERPGMLDF
51 KGKAKWDAWN ELKGNCFNTL PSFGNPSHEI LLRSVRGKDR EQNG

Unformatted sequence string: 94 residues (for pasting into other applications).

Sort by ◉ residue number ○ increasing mass ○ decreasing mass
Show ◉ matched peptides only ○ predicted peptides also

Query	Start – End	Observed	Mr(expt)	Mr(calc)	ppm	M	Score	Expect	Rank	U	Peptide
10671	18 – 33	657.9881	1970.9425	1970.9448	-1.17	0	43	0.00019	1	U	K.TKPADEEMLFIYSHYK.Q

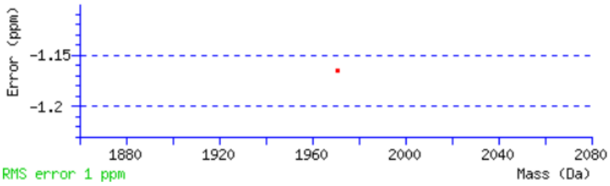

Supplement: Supplementary file 7 — jf1c02016_si_007.pdf [file jf1c02016_si_007.pdf]
