# Supplementary material for: Protein Biomarkers of Bovine Defective Meats at a Glance: Gel-Free Hybrid Quadrupole-Orbitrap Analysis for Rapid Screening
Source: J Agric Food Chem. 2021 Jun 25;69(26):7478–87. doi: 10.1021/acs.jafc.1c02016 (PMC8278482; doi:10.1021/acs.jafc.1c02016)
Supplement: Supplementary file 8 — jf1c02016_si_008.pdf [file jf1c02016_si_008.pdf]

Figure S3A

Mascot Search Results

Peptide View

MS/MS Fragmentation of **GAAQNIIPASTGAAK**
Found in **P10096** in **UP9136_B_taurus**, Glyceraldehyde-3-phosphate dehydrogenase OS=Bos taurus OX=9913 GN=GAPDH PE=1 SV=4

Match to Query 6624: 1368.736132 from(1369.743408,1+) intensity(34430460.0000) scans(6965) rtinseconds(1847.79) index(3668)
Title: QexNORMALhesiOFF.06965.06965.1
Data file QexNORMALhesiOFF.mgf

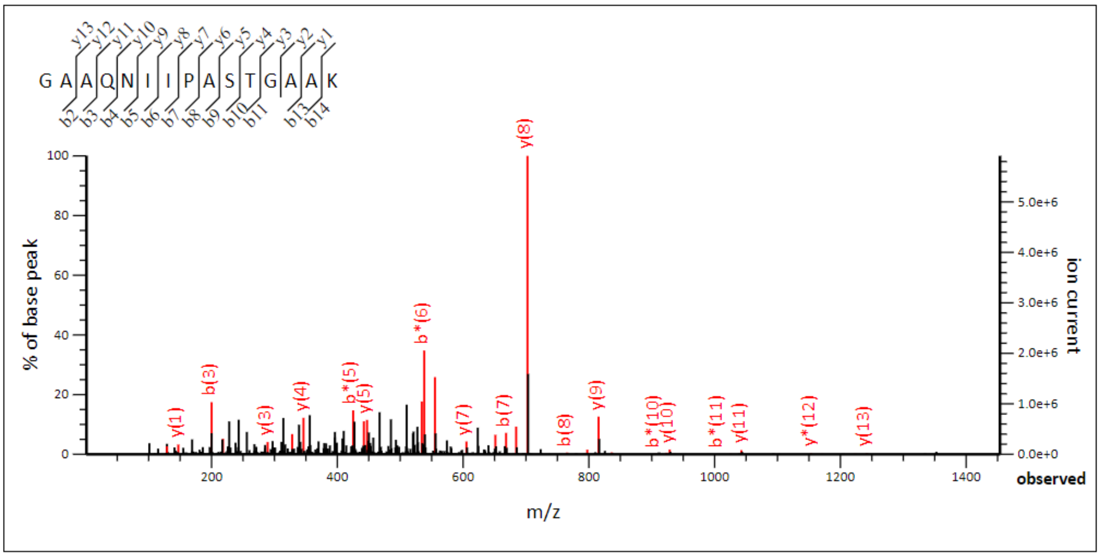

Label all possible matches ○ Label matches used for scoring ◉

Monoisotopic mass of neutral peptide Mr(calc): 1368.7361
Ions Score: 77 **Expect:** 5.6e-08
Matches : 34/68 fragment ions using 81 most intense peaks (help)

#	b	b*	b^0	Seq.	y	y*	y^0	#
1	58.0287			G				15
2	129.0659			A	1312.7219	1295.6954	1294.7114	14
3	200.1030			A	1241.6848	1224.6583	1223.6743	13
4	328.1615	311.1350		Q	1170.6477	1153.6212	1152.6371	12
5	442.2045	425.1779		N	1042.5891	1025.5626	1024.5786	11
6	555.2885	538.2620		I	928.5462	911.5197	910.5356	10
7	668.3726	651.3461		I	815.4621	798.4356	797.4516	9
8	765.4254	748.3988		P	702.3781	685.3515	684.3675	8
9	836.4625	819.4359		A	605.3253	588.2988	587.3148	7
10	923.4945	906.4680	905.4839	S	534.2882	517.2617	516.2776	6
11	1024.5422	1007.5156	1006.5316	T	447.2562	430.2296	429.2456	5
12	1081.5637	1064.5371	1063.5531	G	346.2085	329.1819		4
13	1152.6008	1135.5742	1134.5902	A	289.1870	272.1605		3
14	1223.6379	1206.6113	1205.6273	A	218.1499	201.1234		2
15				K	147.1128	130.0863		1

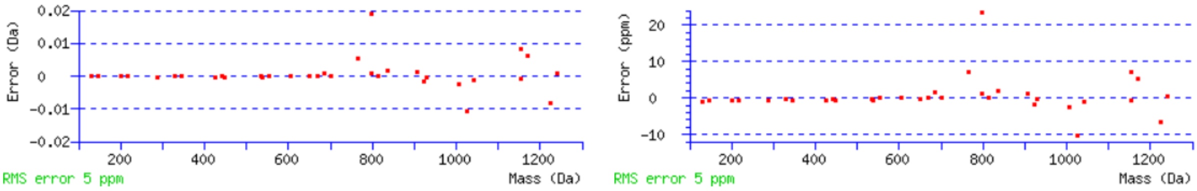

NCBI **BLAST** search of GAAQNIIPASTGAAK
(Parameters: blastp, nr protein database, expect=20000, no filter, PAM30)
Other BLAST web gateways

All matches to this query

Score	Mr(calc)	Delta	Sequence
77.3	1368.7361	0.0000	GAAQNIIPASTGAAK

Figure S3B

Mascot Search Results

Peptide View

MS/MS Fragmentation of **GVFPENFTER**
Found in **A0A3Q1LV21** in **UP9136_B_taurus**, Bridging integrator 1 OS=Bos taurus OX=9913 GN=BIN1 PE=4 SV=1

Match to Query 5373: 1194.566502 from(598.290527,2+) intensity(4694901.5000) scans(7479) rtinseconds(1930.22) index(4835)
Title: QexNORMALhesiOFF.07479.07479.2
Data file QexNORMALhesiOFF.mgf

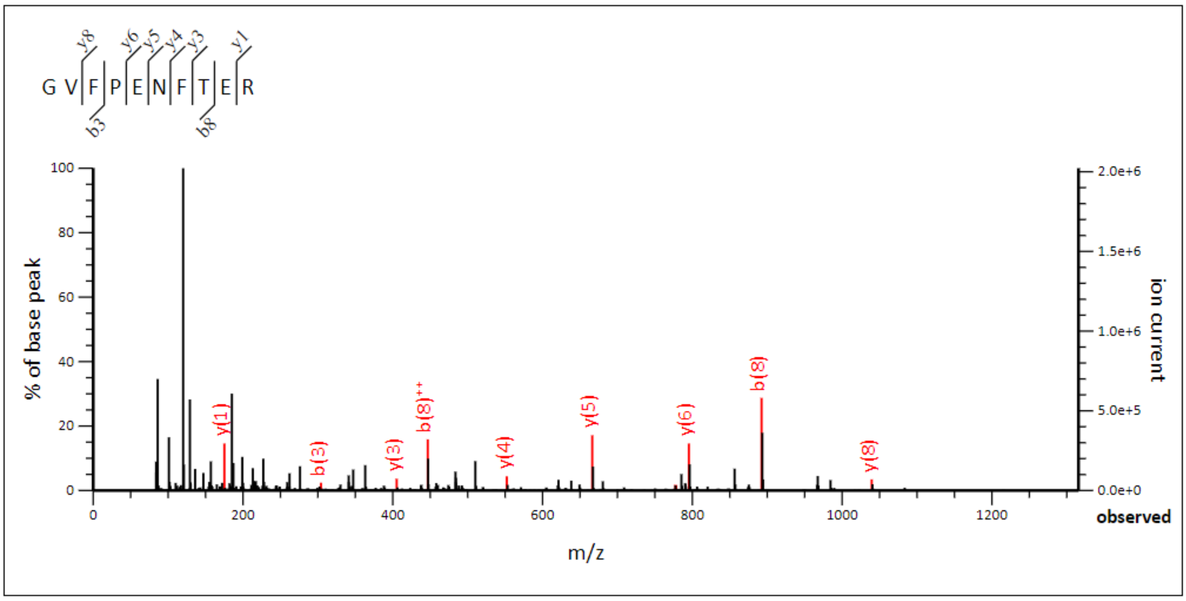

Label all possible matches ○ Label matches used for scoring ◉

Monoisotopic mass of neutral peptide Mr(calc): 1194.5669
Ions Score: 22 **Expect:** 0.031
Matches : 14/88 fragment ions using 52 most intense peaks (help)

#	b	b^{++}	b*	b^{*++}	b^0	b^{0++}	Seq.	y	y^{++}	y*	y^{*++}	y^0	y^{0++}	#
1	58.0287	29.5180					G							10
2	157.0972	79.0522					V	1138.5527	569.7800	1121.5262	561.2667	1120.5422	560.7747	9
3	**304.1656**	152.5864					F	***1039.4843***	520.2458	1022.4578	511.7325	1021.4738	511.2405	8
4	401.2183	201.1128					P	***892.4159***	**446.7116**	875.3894	438.1983	874.4054	437.7063	7
5	530.2609	265.6341			512.2504	256.6288	E	***795.3632***	398.1852	778.3366	389.6719	777.3526	389.1799	6
6	644.3039	322.6556	627.2773	314.1423	626.2933	313.6503	N	***666.3206***	333.6639	649.2940	325.1506	648.3100	324.6586	5
7	791.3723	396.1898	774.3457	387.6765	773.3617	387.1845	F	***552.2776***	276.6425	535.2511	268.1292	534.2671	267.6372	4
8	**892.4199**	**446.7136**	875.3934	438.2003	874.4094	437.7083	T	***405.2092***	203.1082	388.1827	194.5950	387.1987	194.1030	3
9	1021.4625	511.2349	1004.4360	502.7216	1003.4520	502.2296	E	***304.1615***	152.5844	287.1350	144.0711	286.1510	143.5791	2
10							R	***175.1190***	88.0631	158.0924	79.5498			1

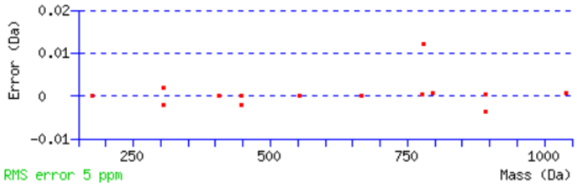

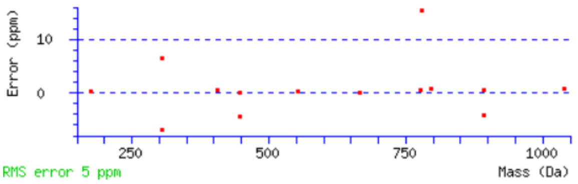

NCBI **BLAST** search of GVFPENFTER
(Parameters: blastp, nr protein database, expect=20000, no filter, PAM30)
Other BLAST web gateways

All matches to this query

Score	Mr(calc)	Delta	Sequence
21.7	1194.5669	-0.0004	GVFPENFTER

Figure S3C

Mascot Search Results

Peptide View

MS/MS Fragmentation of **LLQDFFNGKELNK**
Found in **A0A3Q1LMS5** in **UP9136_B_taurus**, Heat shock cognate 71 kDa protein OS=Bos taurus OX=9913 GN=HSPA8 PE=1 SV=1

Match to Query 7825: 1564.827856 from(783.421204,2+) intensity(2432589.7500) scans(7509) rtinseconds(1925.74) index(4774)
Title: QexDFDhesiOFF.07509.07509.2
Data file QexDFDhesiOFF.mgf

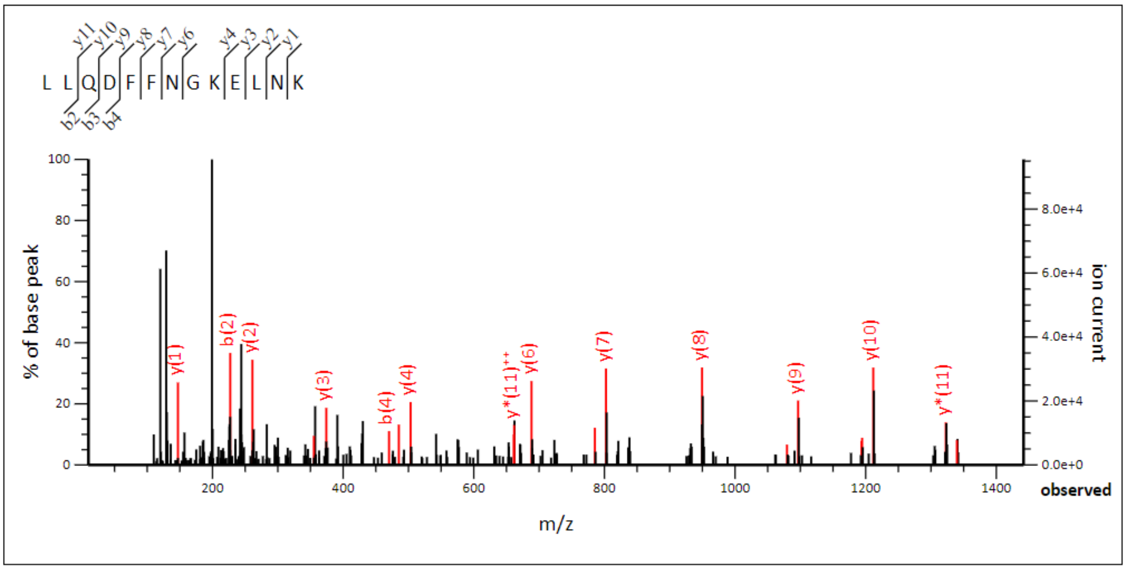

Label all possible matches ○ Label matches used for scoring ◉

Monoisotopic mass of neutral peptide Mr(calc): 1564.8249
Ions Score: 47 **Expect:** 3.7e-05
Matches : 21/128 fragment ions using 52 most intense peaks (help)

#	b	b++	b*	b*++	b0	b0++	Seq.	y	y++	y*	y*++	y0	y0++	#
1	114.0913	57.5493					L							13
2	**227.1754**	114.0913					L	1452.7482	726.8777	1435.7216	718.3644	1434.7376	717.8724	12
3	**355.2340**	178.1206	338.2074	169.6074			Q	***1339.6641***	670.3357	1322.6375	661.8224	1321.6535	661.3304	11
4	**470.2609**	235.6341	453.2344	227.1208	452.2504	226.6288	D	***1211.6055***	606.3064	1194.5790	597.7931	1193.5949	597.3011	10
5	617.3293	309.1683	600.3028	300.6550	599.3188	300.1630	F	***1096.5786***	548.7929	1079.5520	540.2796	1078.5680	539.7876	9
6	764.3978	382.7025	747.3712	374.1892	746.3872	373.6972	F	***949.5102***	475.2587	932.4836	466.7454	931.4996	466.2534	8
7	878.4407	439.7240	861.4141	431.2107	860.4301	430.7187	N	***802.4417***	401.7245	785.4152	393.2112	784.4312	392.7192	7
8	935.4621	468.2347	918.4356	459.7214	917.4516	459.2294	G	***688.3988***	344.7030	671.3723	336.1898	670.3883	335.6978	6
9	1063.5571	532.2822	1046.5306	523.7689	1045.5465	523.2769	K	631.3774	316.1923	614.3508	307.6790	613.3668	307.1870	5
10	1192.5997	596.8035	1175.5732	588.2902	1174.5891	587.7982	E	***503.2824***	252.1448	486.2558	243.6316	485.2718	243.1395	4
11	1305.6838	653.3455	1288.6572	644.8322	1287.6732	644.3402	L	***374.2398***	187.6235	357.2132	179.1103			3
12	1419.7267	710.3670	1402.7001	701.8537	1401.7161	701.3617	N	***261.1557***	131.0815	244.1292	122.5682			2
13							K	***147.1128***	74.0600	130.0863	65.5468			1

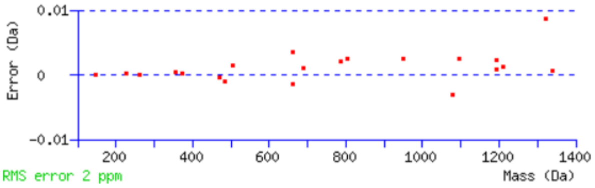

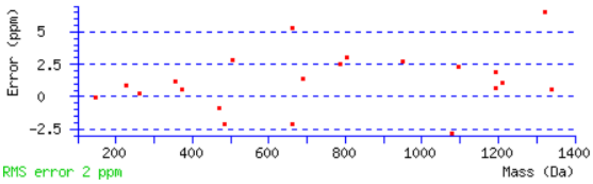

NCBI **BLAST** search of LLQDFFNGKELNK
(Parameters: blastp, nr protein database, expect=20000, no filter, PAM30)
Other BLAST web gateways

All matches to this query

Score	Mr(calc)	Delta	Sequence
47.3	1564.8249	0.0029	LLQDFFNGKELNK

Figure S3D

Mascot Search Results

Peptide View

MS/MS Fragmentation of **TKPADEEMLFIYSHYK**
Found in **A0A3Q1M8I0** in **UP9136_B_taurus**, Acyl-CoA-binding protein OS=Bos taurus OX=9913 GN=DBI PE=1 SV=1

Match to Query 10671: 1970.942466 from(657.988098,3+) intensity(5124570.0000) scans(7310) rtinseconds(1892.54) index(4361)
Title: QexDFDhesiOFF.07310.07310.3
Data file QexDFDhesiOFF.mgf

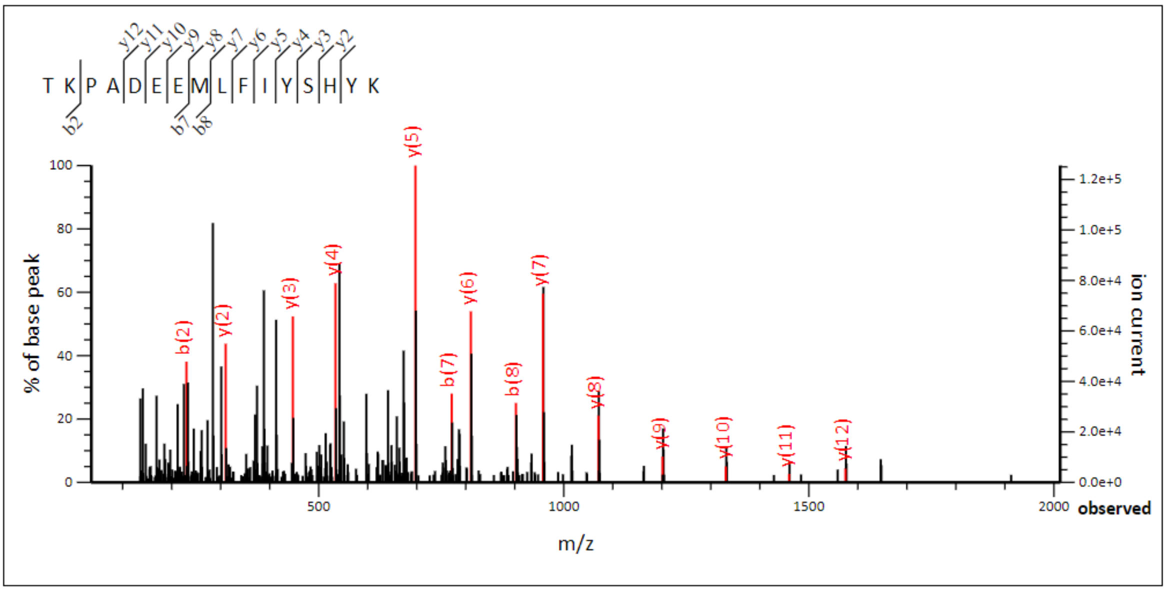

Label all possible matches ○ Label matches used for scoring ◉

Monoisotopic mass of neutral peptide Mr(calc): 1970.9448
Ions Score: 43 **Expect:** 0.00019
Matches : 15/172 fragment ions using 46 most intense peaks (help)

#	b	b^{++}	b*	b^{*++}	b^0	b^{0++}	Seq.	y	y^{++}	y*	y^{*++}	y^0	y^{0++}	#
1	102.0550	51.5311			84.0444	42.5258	T							16
2	**230.1499**	115.5786	213.1234	107.0653	212.1394	106.5733	K	1870.9044	935.9558	1853.8778	927.4426	1852.8938	926.9506	15
3	327.2027	164.1050	310.1761	155.5917	309.1921	155.0997	P	1742.8094	871.9084	1725.7829	863.3951	1724.7989	862.9031	14
4	398.2398	199.6235	381.2132	191.1103	380.2292	190.6183	A	1645.7567	823.3820	1628.7301	814.8687	1627.7461	814.3767	13
5	513.2667	257.1370	496.2402	248.6237	495.2562	248.1317	D	***1574.7196***	787.8634	1557.6930	779.3501	1556.7090	778.8581	12
6	642.3093	321.6583	625.2828	313.1450	624.2988	312.6530	E	***1459.6926***	730.3499	1442.6661	721.8367	1441.6821	721.3447	11
7	**771.3519**	386.1796	754.3254	377.6663	753.3414	377.1743	E	***1330.6500***	665.8286	1313.6235	657.3154	1312.6395	656.8234	10
8	**902.3924**	451.6998	885.3659	443.1866	884.3818	442.6946	M	***1201.6074***	601.3074	1184.5809	592.7941	1183.5969	592.3021	9
9	1015.4765	508.2419	998.4499	499.7286	997.4659	499.2366	L	***1070.5669***	535.7871	1053.5404	527.2738	1052.5564	526.7818	8
10	1162.5449	581.7761	1145.5183	573.2628	1144.5343	572.7708	F	***957.4829***	479.2451	940.4563	470.7318	939.4723	470.2398	7
11	1275.6290	638.3181	1258.6024	629.8048	1257.6184	629.3128	I	***810.4145***	405.7109	793.3879	397.1976	792.4039	396.7056	6
12	1438.6923	719.8498	1421.6657	711.3365	1420.6817	710.8445	Y	***697.3304***	349.1688	680.3039	340.6556	679.3198	340.1636	5
13	1525.7243	763.3658	1508.6978	754.8525	1507.7137	754.3605	S	***534.2671***	267.6372	517.2405	259.1239	516.2565	258.6319	4
14	1662.7832	831.8952	1645.7567	823.3820	1644.7727	822.8900	H	***447.2350***	224.1212	430.2085	215.6079			3
15	1825.8466	913.4269	1808.8200	904.9136	1807.8360	904.4216	Y	***310.1761***	155.5917	293.1496	147.0784			2
16							K	147.1128	74.0600	130.0863	65.5468			1

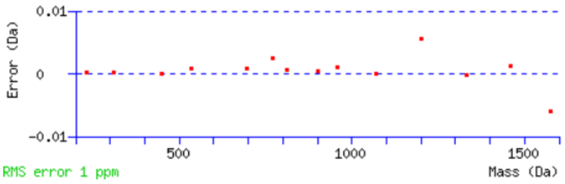

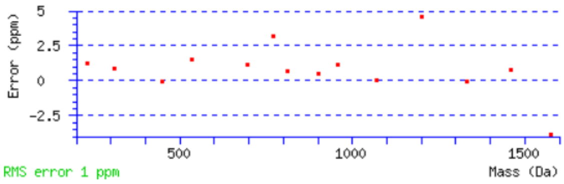

NCBI **BLAST** search of TKPADEEMLFIYSHYK
(Parameters: blastp, nr protein database, expect=20000, no filter, PAM30)
Other BLAST web gateways

All matches to this query

Score	Mr(calc)	Delta	Sequence
43.3	1970.9448	-0.0023	TKPADEEMLFIYSHYK
