# Supplementary material for: Protein Biomarkers of Bovine Defective Meats at a Glance: Gel-Free Hybrid Quadrupole-Orbitrap Analysis for Rapid Screening
Source: J Agric Food Chem. 2021 Jun 25;69(26):7478–87. doi: 10.1021/acs.jafc.1c02016 (PMC8278482; doi:10.1021/acs.jafc.1c02016)
Supplement: Supplementary file 9 — jf1c02016_si_009.pdf [file jf1c02016_si_009.pdf]

MATRIX SCIENCE Mascot Search Results

Peptide View

MS/MS Fragmentation of **VEHTSQGAK**
Found in **F1MJ28** in **UP9136_B_taurus**, Alpha-1,4 glucan phosphorylase OS=Bos taurus OX=9913 GN=PYGM PE=3 SV=2

Match to Query 3955: 955.472692 from(478.743622,2+) intensity(1263351.3750) scans(4039) rtinseconds(1037.24) index(2632)
Title: QexNORMALhesiOFF.04039.04039.2
Data file QexNORMALhesiOFF.mgf

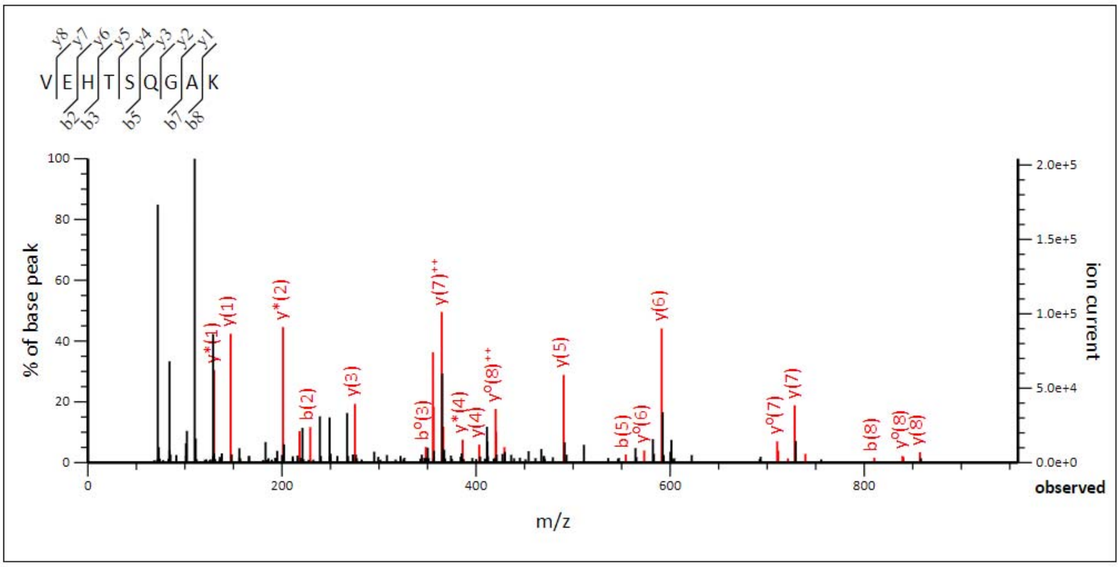

Monoisotopic mass of neutral peptide Mr(calc): 955.4723
Ions Score: 54 **Expect:** 1.5e-05
Matches : 29/76 fragment ions using 54 most intense peaks (help)

#	b	b^{++}	b^{*}	b^{*++}	b^{0}	b^{0++}	Seq.	y	y^{++}	y^{*}	y^{*++}	y^{0}	y^{0++}	#
1	100.0757	50.5415					V							9
2	**229.1183**	115.0628			211.1077	106.0575	E	***857.4112***	**429.2092**	840.3846	420.6959	839.4006	420.2039	8
3	**366.1772**	183.5922			348.1666	174.5870	H	***728.3686***	**364.6879**	711.3420	356.1747	710.3580	355.6826	7
4	467.2249	234.1161			449.2143	225.1108	T	***591.3097***	296.1585	574.2831	287.6452	573.2991	287.1532	6
5	**554.2569**	277.6321			536.2463	268.6268	S	***490.2620***	245.6346	473.2354	237.1214	472.2514	236.6293	5
6	682.3155	341.6614	665.2889	333.1481	664.3049	332.6561	Q	***403.2300***	202.1186	386.2034	193.6053			4
7	**739.3369**	370.1721	722.3104	361.6588	721.3264	361.1668	G	***275.1714***	138.0893	258.1448	129.5761			3
8	**810.3741**	405.6907	793.3475	397.1774	792.3635	396.6854	A	***218.1499***	109.5786	201.1234	101.0653			2
9							K	***147.1128***	74.0600	130.0863	65.5468			1

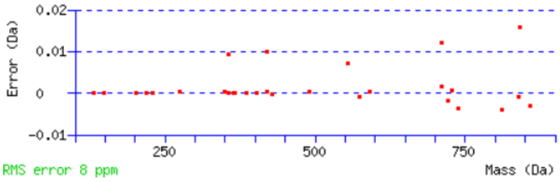

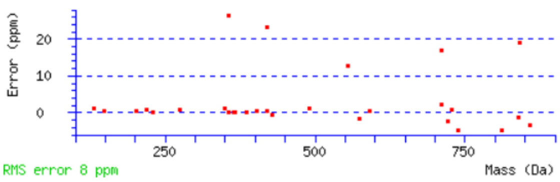

NCBI **BLAST** search of VEHTSQGAK
(Parameters: blastp, nr protein database, expect=20000, no filter, PAM30)
Other BLAST web gateways

All matches to this query

Score	Mr(calc)	Delta	Sequence
53.5	955.4723	0.0004	VEHTSQGAK
1.9	955.4723	0.0004	VQAHAESSK
