# Supplementary figures and images for: Protein Biomarkers of Bovine Defective Meats at a Glance: Gel-Free Hybrid Quadrupole-Orbitrap Analysis for Rapid Screening
Source: J Agric Food Chem. 2021 Jun 25;69(26):7478–87. doi: 10.1021/acs.jafc.1c02016 (PMC8278482; doi:10.1021/acs.jafc.1c02016)

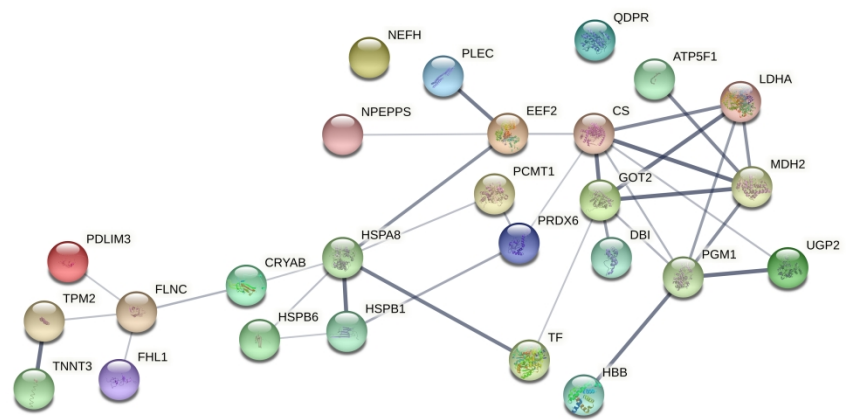

Supplement: Supplementary file 10 — jf1c02016_si_010.pdf [file jf1c02016_si_010.pdf]
