# Supplementary material for: Protein Biomarkers of Bovine Defective Meats at a Glance: Gel-Free Hybrid Quadrupole-Orbitrap Analysis for Rapid Screening
Source: J Agric Food Chem. 2021 Jun 25;69(26):7478–87. doi: 10.1021/acs.jafc.1c02016 (PMC8278482; doi:10.1021/acs.jafc.1c02016)
Supplement: Supplementary file 11 — jf1c02016_si_011.pdf [file jf1c02016_si_011.pdf]

Supplementary file

Samples were studied by merging full-MS^1 and data dependent MS/MS (dd-MS^2) analyses under the following general operating conditions: heated H-ESI option, OFF; full-MS^1 charges of most intense ion to be considered for MS/MS analysis, +1 to +4; microscans, 1; AGC target for full-MS^1 and dd-MS^2 was 1e^6 and 5e^5, respectively; maximum injection time, 120 ms; mass resolution for full-MS^1 and MS/MS analyses was 70000 and 35000 FWHM, respectively; collision HCD energy, 30% (arbitrary units); spray voltage, 4.0 kV; capillary temperature, 250°C; sheath gas, 40 (arbitrary units); auxiliary gas, 0 (arbitrary units); full-MS^1 *m/z* range, 400-2000. Specific dd-MS^2 conditions of analysis were: TopN, 12; dynamic exclusion time, 0.5 min; dynamic exclusion mass width, 10 ppm; repeat count for MS/MS of most intense ion, 2; repeat count duration, 0.5 min; minimum MS/MS ion intensity threshold, 3e^5. Mass tolerance was kept at 5 ppm in both full-MS^1 and dd-MS^2 experiments.
